# *Streptomyces tagetis* sp. nov., a chromomycin producing bacteria isolated from the roots of *Tagetes patula*

**DOI:** 10.3389/fmicb.2024.1361583

**Published:** 2024-03-01

**Authors:** Geeta Chhetri, Myeong Ji Kim, Inhyup Kim, Duc V. H. Tran, Young-Woo Kim, Hyun Woo Kim, Taegun Seo

**Affiliations:** ^1^Department of Life Science, Dongguk University-Seoul, Goyang, Republic of Korea; ^2^College of Pharmacy and Integrated Research Institute for Drug Development, Dongguk University-Seoul, Goyang, Republic of Korea

**Keywords:** *Streptomyces*, *Tagetis patula*, chromomycin, metabolites, melanin, unexplored

## Abstract

A novel halotolerant actinobacterium, designated as RG38^T^, capable of producing black extracellular melanin pigment on SP2 agar, was isolated from the roots of *Tagetes patula*. Comparative analysis of the 16S rRNA gene sequence revealed the highest similarity to *Streptomyces collinus* NBRC 12759^T^ (99.3%). Phylogenetic analysis showed that strain RG38^T^ clustered within the genus *Streptomyces* forming a monophyletic cluster with its close relatives. The average nucleotide identity (ANI), digital DNA–DNA hybridization (dDDH), and amino-acid identity (AAI) values between strain RG38^T^ and related species within the genus *Streptomyces* were below the standard threshold for prokaryotic species delineation. The DNA G + C content of the strain RG38^T^ was determined to be 73.3%. The genome size measured 7,150,598 bp comprising 17 contigs and encompassed 6,053 protein coding genes. AntiSMASH analysis of the whole genome revealed 35 putative biosynthetic gene clusters (BGCs) responsible for various secondary metabolites. Among these clusters, two gene clusters exhibited 100% similarity to the chromomycin A3, albaflavenone, and anthracimycin, respectively. These compounds were reported to possess significant anticancer and antibacterial activities. LC–MS-based analysis, coupled with further isolation studies, confirmed the production of chromomycins A2 (1), A3 (2), and their derivatives, along with their antibiotic activities. These findings underscore the potential of this novel strain as a novel resource for the discovery of diverse antimicrobial compounds. This study is the first to report an antimicrobial compound producing *Streptomyces* species isolated from medicinal plant *T. patula*. Based on a polyphasic study, the strain RG38^T^ isolated from an unexplored habitat with a high potential for new natural products represents a novel species within the genus *Streptomyces*. Accordingly, we propose the name *Streptomyces tagetis* sp. nov. for this novel species, with the type strain is RG38^T^ (=KCTC 49624^T^ = TBRC 15113^T^).

## Introduction

The global challenge of antimicrobial resistance has reached a critical juncture, rendering both common and life-threatening infections increasingly impervious to treatment. The World Health Organization has underscored the urgent necessity for new sources of antibiotics to mitigate the global spread of antibiotic resistance, especially in the context of multi-resistant gram-negative bacteria (Demain, 1999; Kumar et al., 2019). The majority of antibiotic, antitumor, or immunosuppressive compounds of microbial origin discovered to date have been derived from *Streptomyces* (Qin et al., 2011; de Lima Procópio et al., 2012; Ribeiro et al., 2020).

*Streptomyces* species represent a diverse group of gram-positive, filamentous, and spore-producing bacteria, characterized by relatively large genomes, typically measuring 8 to 9 Mbp in size, and a notably high G + C content exceeding 70%. They are well known for their ability to produce antibiotics commonly employed in human medicine, animal health, and agriculture, which they deploy to eliminate competitors. The rhizosphere is an environment teeming with an abundance of bacteria and fungi. These microbes are attracted by the plant exudates secreted through the roots (Pii et al., 2015; Oberhofer et al., 2019; Caracciolo et al., 2021). *Streptomyces* strains confer beneficial effects on plant growth by rendering nutrients available through the degradation of complex biological polymers in the soil or the production of plant growth factors (Ōmura et al., 1982). Members of this genus facilitate host plant growth and mitigate disease symptoms induced by plant pathogens through diverse mechanisms, including the production of bioactive metabolites, employed in direct antagonism against pests and diseases, modulation of host physiological functions, and the induction of host systemic acquired resistance in the host (Qin et al., 2011; Bonaldi et al., 2015). Among these characteristics, a significant common function of *Streptomyces* is their capacity for antibiotic production (de Lima Procópio et al., 2012). This underscores the pivotal role played by the *Streptomyces* genus in bolstering plant defense mechanisms and their widespread recognition for their biocontrol potential. These bioactive compounds are synthesized by biosynthetic gene clusters (BGCs) comprising genes closely arranged within the bacterial genomes (Zhang et al., 1997; Laiple et al., 2009; Naughton et al., 2017; Alam et al., 2022). *Streptomyces* not only produce antibiotics but also yield antifungal, antiparasitic, antiviral, anti-tumoral, and immunosuppressive analogs and other essential secondary metabolites (Alam et al., 2022).

The antimicrobial activity of plant extract of *Tagetes patula* (marigold), a medicinal plant, has been studied as previously investigated (Kashif et al., 2015; Verma et al., 2018; Chandra et al., 2023). However, limited knowledge exists regarding the novel *Streptomyces* strains associated with the roots of the host plant that exhibit antagonistic properties against various pathogenic bacteria. Marigold contains essential oils and high concentrations of flavonoids such as carotene. It functions as an anti-inflammatory agent promoting topical healing and soothing irritated skin. This plant has been highly esteemed by herbal healers for centuries and has been utilized as a medicinal flower to address cuts, sores, and general care among the ethnic population of Nepal. In rural areas of Nepal, where access to government healthcare facilities is challenging, individuals rely on medicinal plants and local healers for their health needs (Ambu et al., 2020). To date, no study has analyzed bacteria isolated from *T. patula* demonstrating antagonistic activity against pathogenic bacteria apart from our previous investigations (Chhetri et al., 2022).

This study aimed to explore the potential of a novel *Streptomyces* strain, designated as RG38^T^, which was isolated from an unexplored habitat. The genome of this strain was sequenced and examined for its secondary metabolites. It was observed that *Streptomyces* strain RG38^T^ exhibits substantial antibacterial activity against *Staphylococcus epidermidis* KACC 13234, *Bacillus subtilis* KACC 16747, *Micrococcus luteus* KACC 13377, and *Staphylococcus aureus* ATCC 6538. Furthermore, this strain possesses a substantial number of BGCs in its genome, indicating its capacity to produce bioactive compounds which are identified as chromomycin A2 (1) and A3 (2). Chromomycins belong to the aureolic acid family of antitumor compounds and were initially isolated from *Streptomyces griseus* No. 7 (ATCC No. 13273) (Kamiyama and Kaziro, 1966). Chromomycins, olivomycins, chromocyclomycin, mithramycin, UCH9, and durhamycin A belong to the class of antitumor compounds known as aureolic acids (Alam et al., 2022).

In pursuit of the discovery of novel culturable and beneficial bacteria from unexplored habitats such as roots of *T. patula*, our team isolated more than 100 bacterial species related to Actinobacteria. We exclusively registered novel species in GenBank and have already published a few articles demonstrating the antimicrobial activity and rice plant growth promoting ability of *Chryseobacterium tagetis* RG1^T^ (Chhetri et al., 2022). Among these novel species, a few species did not exhibit antimicrobial activity, but genomic data suggested their involvement in plant growth promoting activities. Thus, our study suggests the need for further investigation into unexplored habitats, such as *T. patula*, particularly in South Korea where people are unfamiliar even with its name to explore beneficial bacteria.

## Materials and methods

### Isolation, cultivation, and preservation

We encountered limited previous research pertaining to bacterial diversity in the roots of marigold plants. These unexplored environments present promising prospects for the discovery of rare actinomycetes that are believed to be rich sources of novel bioactive compounds. Therefore, to investigate the analysis of beneficial and culturable bacterial community in the roots of these plants, we collected samples from the garden of Dongguk University, Ilsan, Republic of Korea (37° 40′ 26.4” N 126° 48′ 20.88″ E) (Supplementary Figure S1). To prepare the samples, we removed the roots from the soil. Subsequently, a thorough washing with sterile water was conducted to remove any external soil from the surface. The root surface was disinfected with 70% ethanol for 1 min and washed several times with sterile distilled water again. The surface-sterilized roots were then pulverized using a ceramic mortar. Serial dilutions using 0.85% NaCl saline were made, and aliquots of 0.1 mL from decimal dilution (10^−3^–10^−7^) were plated on Reasoner’s 2 agar (R2A) (Difco). After 1 week of incubation of the inoculated plates at 30°C, isolates were obtained. Fourty-eight different colonies were selected according to their different morphology and color. Each individual colony was purified by transferring to them onto new R2A, nutrient agar (NA), and tryptic soy agar (TSA) plates. Subsequently, these purified colonies were sent to Bionics (Daejeon, Republic of Korea) for 16S rRNA gene analysis. The isolates were cultivated on R2A agar at 30°C for 5 days and simultaneously maintained in 50% glycerol at −80°C.

### Phylogenetic and genome annotation analysis

Bacterial DNA was extracted using the TaKaRa MiniBEST Bacteria Genomic DNA extraction Kit version 3.0 (TaKaRa), following the manufacturer’s instructions. Small subunit 16S rRNA gene fragments were amplified with universal bacterial primers 27F, 518F, 805R, and 1492R; the resulting PCR products were commercially sequenced (Solgent, Korea). The 16S rRNA gene sequence identity similarities between strain RG38^T^ and other type strains were determined, and phylogenetic relationships were analyzed using the EzBioCloud server (Kim et al., 2012). Multiple sequence alignments were performed with the ClustalW program (Thompson et al., 1997). Phylogenetic and molecular evolutionary analyses were conducted using MEGA version 7.0 (Kumar et al., 2016). Clustering was determined using the neighbor-joining (NJ), maximum-likelihood (ML), and maximum-parsimony (MP) algorithms. The distances for the NJ, ML, and MP trees were calculated according to the two-parameter model of Kimura (1980), and the bootstrap values were established based on 1,000 replicates (Felsenstein, 1985).

The strain RG38^T^ was subjected to whole-genome shotgun sequencing at Macrogen (Republic of Korea) using the Illumina HiSeq 2,500 platform with a 150-bp × 2 paired-end kit. Subsequently, the data were assembled using the SPAdes analysis v.3.10.1 at Macrogen (Seoul, Republic of Korea). After assembly, the locations of protein genes were predicted, and their functions were annotated. Prokka was employed for location prediction, while BLAST and evolutionary genealogy of genes: Nonsupervised Orthologus Groups (eggNOG) 4.5 database (Huerta-Cepas et al., 2017) were used for functional determination and identification of assembled sequences against the nucleotide and protein sequence databases. The genomes of the reference strains were retrieved from the NCBI database and used as references for digital DDH and ANI analysis of the query genome of strain RG38^T^. The estimated DDH values were calculated by the recommended formula 2 (identities/HSP length) using the Genome-to-Genome Distance Calculator (GGDC)^1^ (Meier-Kolthoff et al., 2013), and ANI values were from EZ Biocloud platform^2^ (Yoon et al., 2017a). Average amino acid identity (AAI) values were calculated from protein sequences by using an online AAI calculator^3^. Two-way AAI analysis was used. Genomic circular feature map was constructed using CGView server (Grant and Stothard, 2008). To evaluate the intergenomic distances between genome sequences of the strain RG38^T^ and its close strains belonging to the phylogenetically closest *Streptomyces* species, the FastANI values were also determined (Jain et al., 2018). The DNA G + C content of the strain RG38^T^ was calculated based on whole-genome sequence. CheckM bioinformatics tool was used to assess genome contamination and completeness^4^ of the strain RG38^T^ (Parks et al., 2015). To enhance the phylogenetic classification and gain a better understanding of the relationships between the novel isolate and closely related species, phylogenomic trees were constructed based on an up-to-date bacterial 92 core gene set (UBCG) (Na et al., 2018). The draft genome of the strain RG38^T^ was then analyzed for the presence of BGCs using antiSMASH 5.0 online software (Naughton et al., 2017).

### Phenotypic, morphological, and chemotaxonomic features

Comparative studies were conducted for all strains in triplicate on International Streptomyces Project (ISP) 2 media (ISP2) at 30°C. For morphology, cells were grown on ISP2 medium for 7 days at 30°C. Subsequently, cells were fixed in 2.5% (v/v) glutaraldehyde and stored for 4 h at 4°C, and fixing agent was removed by rinsing with PBS (phosphate buffer saline). Dehydration was achieved through successive washes with 30, 50, 60, 70, 80, and 90% (v/v) and absolute ethanol. Then, the cells were subjected to critical point dehydration at room temperature followed by mounting on a stub with a carbon disk. After overnight drying, a platinum coating was applied using a scanning electron microscopy (SEM) coating unit (15 nm; EM ACE200, Leica, Wetzlar, Germay). Images were analyzed using field emission-scanning electron microscopy (FE-SEM) analysis.

Cultural characteristics for the strain were determined after incubation at 30°C for 2 weeks using ISP2 media, Bennett’s agar (BA), Nutrient agar (NA), Reasoner’s 2 agar (R2A), Marine agar (MA), Luria bertani agar (LBA), and Tryptic soy agar (TSA; Difco). Growth was tested at different temperatures (4, 10, 15, 20, 25, 30, 35, 40, 45, and 50°C). NaCl tolerance was determined on ISP2 medium supplemented with 1–16% NaCl (w/v, with an interval of 1% w/v) at 30°C for 14 days. The pH range for growth was determined by cultivation at 30°C in ISP2 broth adjusted to pH 4–10 (at pH 1 unit intervals) before sterilization with citrate/NaH_2_PO4 buffer (pH 4.0–5.0), phosphate buffer (pH 6.0–8.0), and Tris buffer (pH 9.0–10.0) as described previously (Kim et al., 2020; Chhetri et al., 2021a). The pH values were verified after autoclaving. Cellular fatty acid profiles and quinones were not analyzed in this study, owing to the revised guidelines of the International Code of Nomenclature of Prokaryotes or IJSEM for taxonomic descriptions. According to these updated guidelines, chemotaxonomic analyses, such as polar lipid and fatty acid analysis, are no longer obligatory for the characterization of novel bacterial species. They are only mandated for novel genera^5^.

Biochemical tests were conducted using the API 20NE and API ZYM strips (bioMérieux), following the manufacturer’s instructions. Additionally, Tween (20, 40, and 80), casein, starch, CM cellulose, and chitin were performed as previously described (Kim et al., 2019; Chhetri et al., 2021b, 2023).

### Antimicrobial activity on agar media

The strain RG38^T^ was cultured in R2A, NA, TSA, BA, MA, LBA, and ISP2 media at 30°C to determine the best optimal medium for antibiotic production. Pathogenic bacteria *Staphylococcus epidermidis* KACC 13234, *Staphylococcus aureus* ATCC 6538, *Xanthomonas campestris* pv. *campestris* KACC 10377, *Bacillus subtilis* KACC 16747, *Micrococcus luteus* KACC 13377, *Botrytis cinerea* KACC 40573, and *Pantoea agglomerans* KACC 10054 were grown in Tryptic soya broth (TSB) at 37°C. Subsequently, after a 12-h incubation period, 80 μL aliquots of each pathogenic bacterium were individually spread onto NA plates. Following this, 4-day-old agar blocks of the strain RG38^T^ were transferred onto plates that had been pre-inoculated with the pathogenic bacteria. The plates were then incubated at 30°C for 24 h, and the activity was quantified by measuring the inhibition zone in millimeters surrounding the agar block (Maiti et al., 2020).

### Extraction and purification of bioactive compounds

The agar extraction method, employed in this study, was adapted from a previous study (Carr et al., 2010). In brief, 30 large Petri dishes (150 mm x 15 mm) were used. Strain RG38^T^ was spread on NA plates and then incubated at 30°C. After 4 days of incubation, the agar in the plates was chopped into small pieces. These agar pieces were collected in two separate 2 L bottle, into which ethyl acetate (1 L) was added to facilitate the absorption of agar pieces. The bottles were rotated at 150 rpm in room temperature and left for 1 day. The resultant ethyl acetate solution was filtered using Whatman™ filter paper 42 (150 mm; GE healthcare UK). The filtered solution was centrifuged to remove the remnants at 5,000 rpm for 20 min at 4°C. The organic layer was collected and completely concentrated using a rotary evaporator at 40°C as described previously (Chhetri et al., 2022). The remaining residue of the crude product was dissolved in 500 μL methanol, and subsequently, 15 μL was applied to a sterile paper disk (6 mm, Whatman). The antimicrobial activity against pathogenic bacteria was assessed, and the zone of inhibition was measured. The extract in methanol was concentrated using a vacuum evaporator and was analyzed by reverse-phase high-performance liquid chromatography (HPLC) using solvents A (H_2_O) and B (acetonitrile) (Agilent 1,260, C18, 3.5 μm, 4.6 × 100 mm, the detection wavelengths were 254 and 280 nm). Major peaks were isolated and purified by HPLC on a gradient comprised of solvents A (H_2_O) and B (acetonitrile): 40–75% B (0–12 min), 75–100% B (12–16 min), 100% B (16–30 min), flow rate: 3 mL/min (Agilent 1,260, SB-C18, 5 μm, 9.4 × 250 mm). Chromomycins A2 (1) and A3 (2) were isolated and purified by HPLC on a gradient comprised of solvents A (H_2_O) and B (acetonitrile): 40–75% B (0–12 min), 75–100% B (12–16 min), 100% B (16–30 min), flow rate: 3 mL/min (Agilent 1,260, SB-C18, 5 μm, 9.4 × 250 mm). Solvents were evaporated under a vacuum using Scanvac Concentrator. Dried pure products were stored at −20°C. Next day, each fraction was tested for antimicrobial activity using 6 mm disk (Whatman). Active fraction obtained was further chromatographed using methanol as a mobile phase and obtained single peak, which indicated the purity of compound. At last, lyophilization of purified compound was performed. NMR spectra of isolated compounds were acquired on a Varian 400 (Varian, Palo Alto, CA, USA) at 25°C.

### UPLC-QTOF-MS/MS-based metabolite profiling

The lyophilized crude extract from the strain RG38^T^ was dissolved in LC–MS grade methanol through sonication and filtered with a 0.2 μm regenerated cellulose (RC) syringe filter (GVS filter technology, IN, USA) with a concentration of 2 mg/mL. The UPLC-QTOF-MS/MS data were obtained using the Waters Acquity UPLC system (Milford, MA, USA), comprising a binary solvent delivery system, an autosampler, and a photo diode array (PDA) detector. The UPLC column utilized was the Waters Acquity UPLC BEH C18 (150 mm × 2.1 mm, 1.7 μm). The mobile phase was 20 mM formic acid in water (A) and acetonitrile (B), with the following gradient: 10–90% B (0–14 min, v/v). The flow rate was set at 300 μL/min, and the injection volume was 2.0 μL. The autosampler and column oven were kept at 15°C and 40°C, respectively. Mass spectrometry experiments were conducted using a Waters Xevo G2 QTOF mass spectrometer (Waters MS Technologies, Manchester, UK), which was connected to the UPLC system through an electrospray ionization (ESI) interface. The ESI condition was set as follows: negative ion mode, capillary voltage 2.5 kV, cone voltage 40 V, source temperature 120°C, desolvation gas temperature 350°C, cone gas flow 50 L/h, and desolvation gas flow 800 L/h. The ion acquisition rate was set at 0.2 s. Data were centroided during acquisition using an independent reference lock-mass ion via the LockSpray™ interface to ensure accuracy and precision. Leucine enkephalin (*m/z* 554.2615 in negative mode) was used at a concentration of 200 pg/μl with an infusion flow rate of 5 μL/min. The acquired data were analyzed using a feature-based molecular networking analysis, which is available on the GNPS web platform^6^ with a spectral preprocessing by MZmine3 software. All the results and parameters can be accessed with the GNPS job id for molecular network analysis^7^ and enhanced analysis^8^.

## Results and discussion

### Phylogenetic and genomic analysis of novel strain RG38^T^

**Figure 1:**
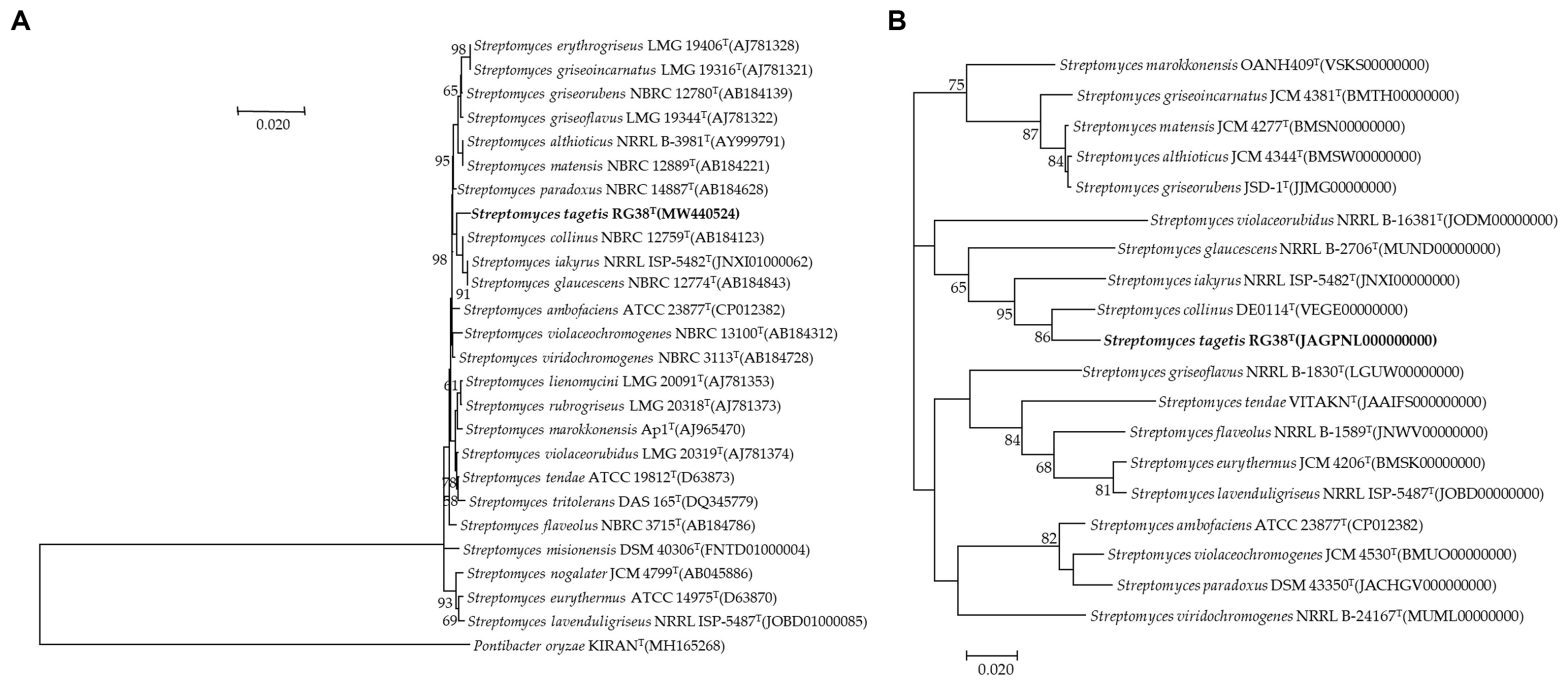
Neighbor-joining tree based on 16S rRNA gene sequences showing the relationship between strain RG38^T^ and related species. *Pontibacter oryzae* KIRAN^T^ (MH165268) was used as an out-group. Bootstrap values (based on 1,000 replications) greater than 50% are shown at branch points. Bar, 0.020 substitutions per nucleotide position **(A)**. Phylogenomic tree of strain RG38^T^ and closely related strains based on core genomes was constructed using UBCG, all genomes of 19 related strains were available on NCBI GenBank. GenBank accession numbers are shown in parentheses. Bootstrap analysis was carried out using 100 replications. Percentage bootstrap values (>50%) are given at branching points. Bar, 0.020 substitution per position **(B)**.

**Figure 2:**
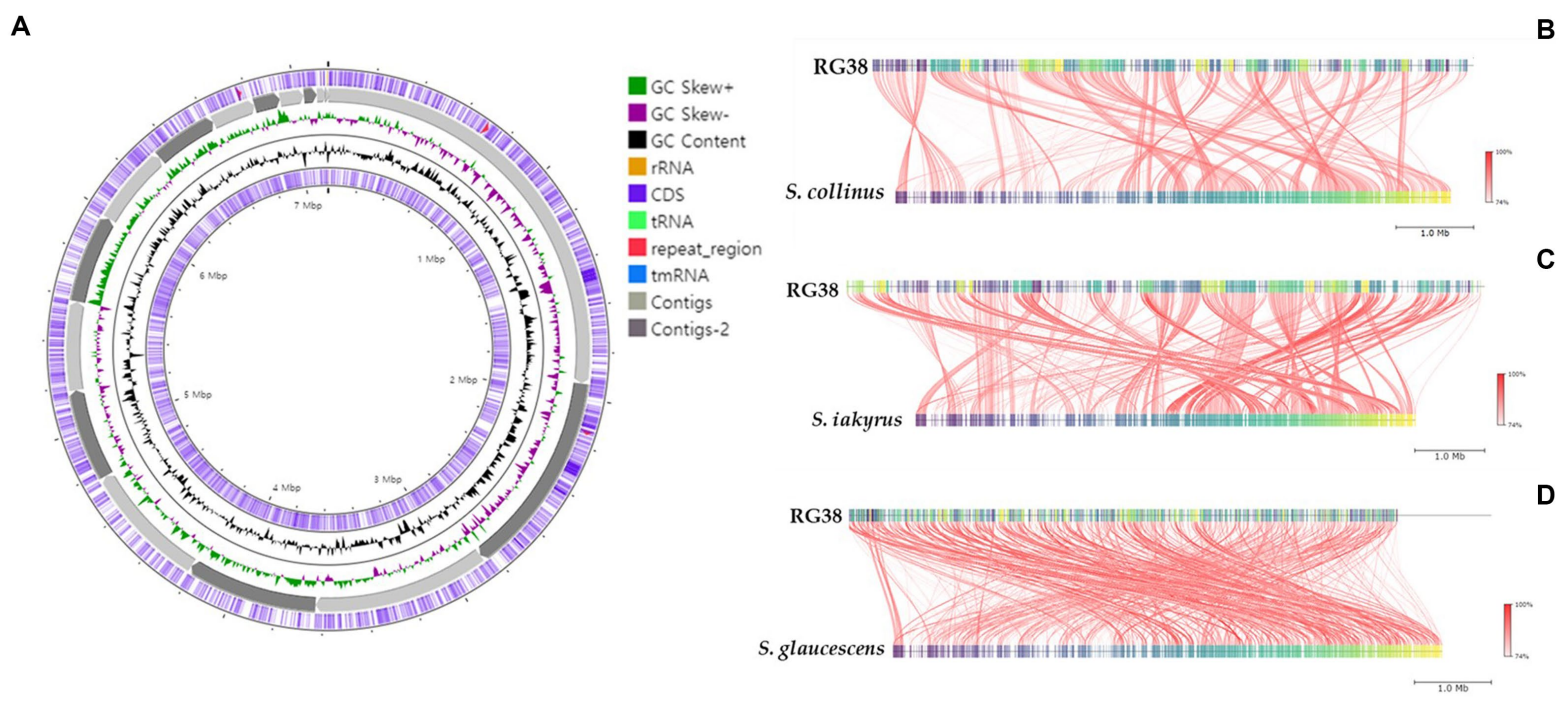
Graphical genome map of strain RG38^T^. RNA genes (tRNAs dark blue, rRNAs light green, and tmRNA red), GC content, and GC skew **(A)**. Illustration of FastANI’s work-flow for computing ANI between a query genome (strain RG38^T^) and a reference genome (*S. collinus* NBRC 12759^T^) **(B)**, *S. iakyrus* NRRL ISP-5482^T^ **(C)** and *S. glaucescens* NBRC 12774^T^ **(D)**. Each red line segment denotes a reciprocal mapping between the query and reference genomes, indicating their evolutionary conserved regions.

In the EzBioCloud analysis, 16S rRNA gene sequences indicated that the closest relatives of the strain RG38^T^ were *Streptomyces collinus* NBRC 12759^T^ (99.3%), *S. violaceochromogenes* NBRC 13100^T^ (99.2%), *S. viridiochromogenes* NBRC 3113^T^ (99.1%), *S. griseoflavus* LMG 19344^T^ (99%), and *S. paradouxus* NBRC 14887^T^ (99%). The 16S rRNA sequence similarity values between strain RG38^T^ and other species ranged from 98.9 to 98.3%. The NJ tree showed the grouping of the strain RG38^T^ within the genus *Streptomyces* and clusters with *S. collinus* NBRC 12759^T^, *S. iakyrus* NRRL ISP-5482^T^, and *S. glaucescens* NBRC 12774T (Figure 1A). Similar results were also obtained in the ML and MP trees (Supplementary Figures S2, S3). Based on the phylogenetic trees, *S. collinus* NBRC 12759^T^, *S. iakyrus* NRRL ISP-5482^T^, and *S. glaucescens* NBRC 12774^T^ were selected to compare the ANI, dDDH, and AAI and characteristics of the strain RG38^T^ with those of the type strains in the genus *Streptomyces*. The selected reference strains were purchased from the KCTC (Korean Collection for Type Cultures) and JCM (Japan Collection of Microorganisms). The draft genome of the strain RG38^T^ contained 19 contigs, with a length of 7.15 Mb and an N50 length of 781,243 bp. A total of 6,289 genes were predicted, which included 6,053 protein-coding genes and 68 tRNA genes. The ANI threshold for species demarcation is recommended to be 95–96%, and the ANI values between the strain RG38^T^ and its phylogenetically closest neighbors were ≤ 84.7% (Table 1). The dDDH values between the strain RG38^T^ and other members of *Streptomyces* genus were ≤ 28.8%, which was lower than the 70% species threshold recommended for species delineation (Meier-Kolthoff et al., 2013; Yoon et al., 2017b). In addition, the tight association of strain RG38^T^ with its closely related strains was supported by the results of phylogenomic analyses. The strain RG38^T^ has AAI values ranging from 68.7 to 81.6% with the all reference genomes, which exceeds the AAI criteria for genus delineation (65%). AAI values between strain RG38^T^ and other species of *Streptomyces* are shown in Table 1. These results are all below the accepted cutoff values for species delineation, indicating that the strain RG38^T^ should be considered and representing a new species of the genus *Streptomyces*. The phylogenomic tree revealed that the strain RG38^T^ formed an independent cluster with *S. collinus* NBRC 12759^T^, *S. iakyrus* NRRL ISP-5482^T^, *S. glaucescens* NBRC 12774^T^, and *S. griseoflavus* NRRL B-1830^T^ with high topology (Figure 1B), demonstrating that the strain RG38^T^ represents a novel species within the genus *Streptomyces*. The CheckM result showed that the genome completeness was 99%, and the contamination level was 0.85%. The DNA G + C content was 73.3%. Genomic circular feature map was constructed using CGView server is shown in Figure 2A. The FastANI values between the strain RG38^T^ and its three reference strains *S. collinus* NBRC 12759^T^, *S. iakyrus* NRRL ISP-5482^T^, and *S. glaucescens* NBRC 12774^T^ were 81.4, 82.6, and 82.7%, which are lower than the 95–96% cutoff values previously proposed for species delimitation (Jain et al., 2018). Each red line segment denotes a reciprocal mapping between the strain RG38^T^ and reference genomes, indicating their evolutionary conserved regions (Figures 2B–D).

**Table 1:** ANI, dDDH, and AAI among strain RG38^T^ and other closely related *Streptomyces* members.

Strains	Accession no	RG38^T^ (%)		
		ANI	dDDH	AAI
*Streptomyces collinus* DE0114^T^	VEGE00000000	81.4	25.1	76
*Streptomyces violaceochromogenes* JCM 4530^T^	BMUO00000000	82.6	26.1	78
*Streptomyces viridochromogenes* NRRL B-24167^T^	MUML00000000	82.2	25.9	77.8
*Streptomyces griseoflavus* NRRL B-1830^T^	LGUW00000000	78.1	22.2	68.7
*Streptomyces paradoxus* DSM 43350^T^	JACHGV000000000	82.6	26.2	78.1
*Streptomyces ambofaciens* ATCC 23877^T^	CP012382	84.7	29	81.6
*Streptomyces althioticus* JCM 4344^T^	BMSW00000000	82.5	25.9	78.6
*Streptomyces flaveolus* NRRL B-1589^T^	JNWV00000000	82	25.6	76.7
*Streptomyces matensis* JCM 4277^T^	BMSN00000000	82.5	26	78.5
*Streptomyces violaceorubidus* NRRL B-16381^T^	JODM00000000	84.5	28.8	81
*Streptomyces griseorubens* JSD-1^T^	JJMG00000000	82.6	26.3	78.5
*Streptomyces glaucescens* NRRL B-2706^T^	MUND00000000	82.7	26.5	77.4
*Streptomyces tendae* CS113^T^	JAAIFS000000000	84.4	28.7	81.2
*Streptomyces iakyrus* NRRL ISP-5482^T^	JNXI00000000	82.6	26.1	78
*Streptomyces eurythermus* JCM 4206^T^	BMSK00000000	82	25.6	76.1
*Streptomyces lavenduligriseus* NRRL ISP-5487^T^	JOBD00000000	82	25.5	76.3
*Streptomyces griseoincarnatus* JCM 4381^T^	BMTH00000000	82.4	25.9	78.3
*Streptomyces marokkonensis* OANH409^T^	VSKS00000000	82.9	26.5	78.9

**Figure 3:**
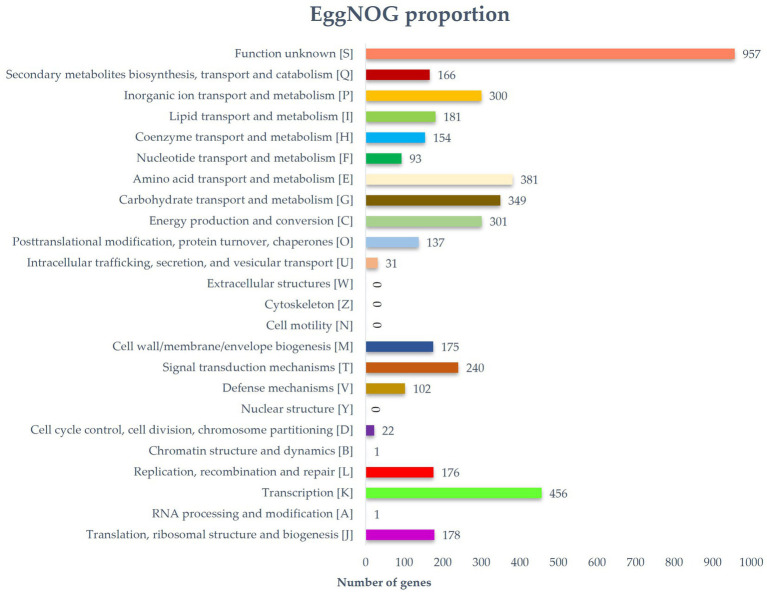
Distribution of genes based on the 24 general eggNOG functional categories of strain RG38^T^.

Predicted proteins were annotated by blasting the eggNOG database. In total, 6,115 out of 6,289 protein coding genes were classified into 24 functional categories based on the eggnog–Mapper using precomputed cluster and phylogenies from the eggnog database as previously described. The following four top categories (unknown functions were skipped) were classified: transcription (456), carbohydrate transport and metabolism (349), and inorganic ion transport and metabolism (300) (Figure 3). Thirty-five BGCs were detected in the genome of the strain RG38^T^. These biosynthetic gene clusters belonged to several cluster categories, noticeably T2PKS, oligosaccharide, phosphonate, linear azol(in)e-containing peptides (LAP), other unspecified ribosomally synthesized and post-translationally modified peptide product (RiPP)-like cluster, terpene, non-ribosomal peptide synthases (NRPS), siderophore, lanthipeptide-i, lanthipeptide-ii, transAT-PKS, betalactone, indole, butyrolactone, linaridin, melanin, ectoine, and lanthipeptide-iv. Interestingly, the genome of the strain RG38^T^ comprised the gene clusters revealing a low similarity percentage (<20%) to Versipelostatin (5%), Herboxidiene (4%), Vazabitide A (19%), and Caniferolide A/ B/C/ D (4%). Thus, they might be involved in the biosynthesis of new compounds. Therefore, it is implied that the strain RG38^T^ is a potential species for producing novel competent compounds. Among them, herboxidiene is a compound isolated from *Streptomyces chromofuscus* strain A7847 and exhibits antitumor activity by suppressing the growth of tumor cells through interfering with the splicing of pre-mRNA coding for cell cycle regulation proteins in our body, and this activity makes herboxitriene a valuable starting point for the development of anticancer drug (Maiti et al., 2020). Moreover, six secondary metabolite biosynthesis gene clusters showed 100% similarities to known biosynthetic gene clusters: T2PKS, oligosaccharide, terpene, transAT-PKS, ectoine, and lantipeptide class iv biosynthetic gene cluster (100%). These gene clusters may be involved in the production of the secondary metabolites in RG38^T^. All bio-clusters found in the genomes of the strain RG38^T^ is presented in Table 2. Gene cluster for geosmin, which is responsible for earthy smell of the strain RG38^T^, was also found.

**Table 2:** Secondary metabolite biosynthesis gene clusters identified in the genome of the strain RG38^T^ with antiSMASH (manually curated).

Metabolite class (type)	Location (nt)	Clusters	Most similar known biosynthetic gene cluster (Percentage of similarity)	(%)
T2PKS	147,466–219,939	2	Chromomycin A3	100
	285,713 - 374,281		Spore pigment	66
Oligosaccharide	147,466–219,939	1	Chromomycin A3	100
Phosphonate	293,972 - 358,530	1	Dehydrophos	35
LAP	293,972 - 358,530	1	Dehydrophos	35
thiopeptide	293,972 - 358,530	1	Dehydrophos	35
RRE-containing	293,972 - 358,530	1	Dehydrophos	35
RiPP-like	507,011 - 518,271	2	-	-
	1,691,581 - 1,701,796		Informatipeptin	42
Terpene	530,371 - 550,743	5	Geosmin	100
	1,084,332 - 1,110,388		Hopene	92
	1,662,287 - 1,683,318		Versipelostatin	5
	566,242 - 630,768		Carotenoid	54
	399,515 - 420,594		Albaflavenone	100
NRPS	574,731 - 620,282	8	CDA1b / CDA2a / CDA2b / CDA3a / CDA3b / CDA4a / CDA4b (Ca + −dependent lipopeptide)	5
	1,426,743 - 1,516,420		Cysteoamide	18
	147,207 - 210,648		Herboxidiene	4
	446,669 - 495,414		Coelichelin	81
	566,242 - 630,768		-	-
	727,279 - 774,508		Streptobactin	64
	122,174- 185,958		Vazabitide A	19
	130,102 - 184,189		Caniferolide A/ B/C/ D	4%
Siderophore	656,754 - 670,047	1	-	-
Lantipeptide class i	147,207 - 210,648	2	Herboxidiene	4
	145,629 - 171,957		-	-
Lantipeptide class ii	147,207 - 210,648	1	Herboxidiene	4
transAT-PKS	318,727 - 407,049	1	Anthracimycin	100
Betalactone	416,391 - 440,650	2	-	-
	285,713 - 374,281		-	-
Indole	678,989 - 700,113	1	5-isoprenylindole-3-carboxylate β-D-glycosyl ester	33
Butyrolactone	818,080 - 829,021	1	-	-
Linaridin	66,758 - 87,754	1	Legonaridin	66
Melanin	311,208–321,753	1	Melanin	60
Ectoine	75,206 - 85,604	1	Ectoine	100
Lantipeptide class IV	145,977 - 168,661	1	Venezuelin	100

### Morphological analysis

**Figure 4:**
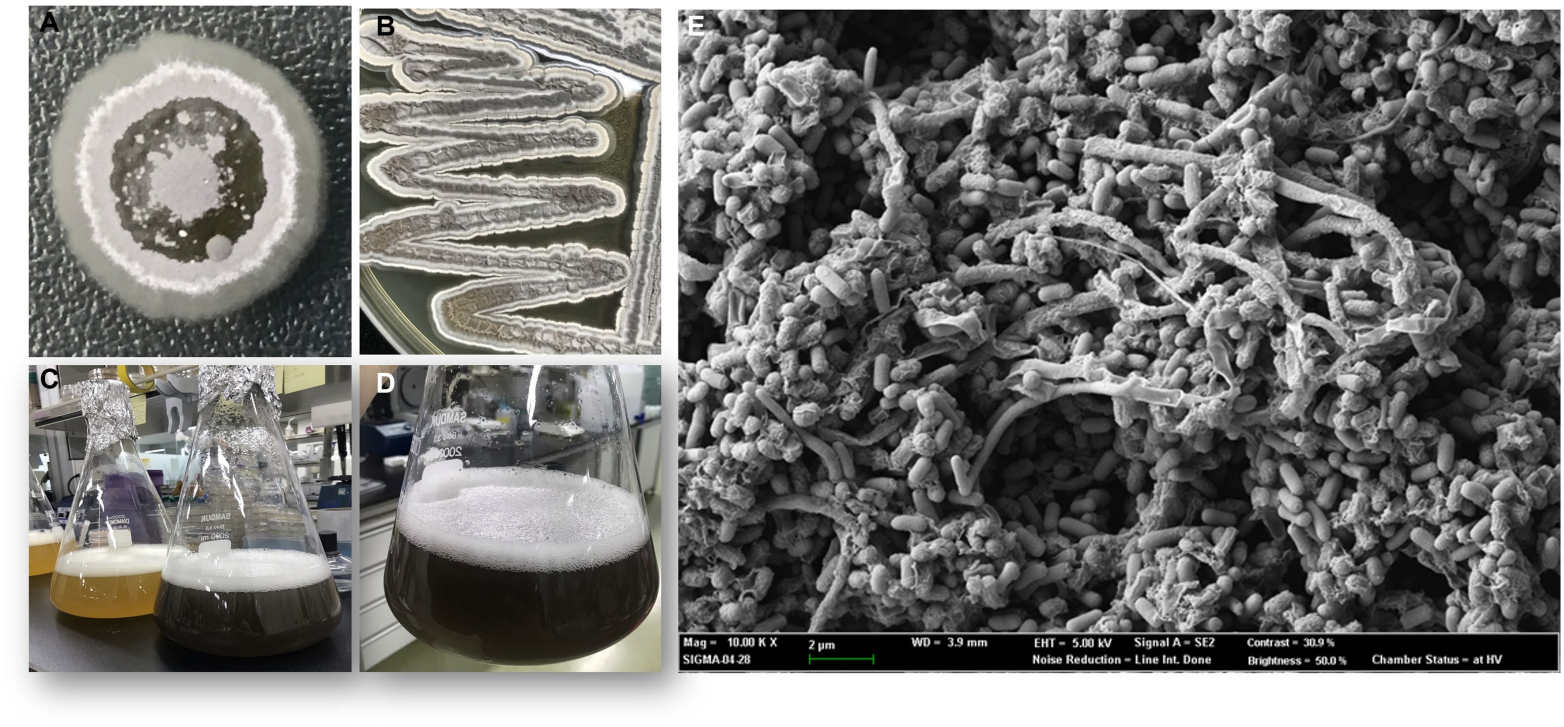
Colony morphology of strain after 5 days of growth at 30°C **(A,B)**. Melanin pigment was not found when they were grown in R2A broth **(C)**. Production of melanin, only in ISP2 media and broth by strain RG38^T^ at 30°C after 7 days of incubation **(A,C,D)**. Scanning electron microscope images of the strain RG38^T^ after 5 days of incubation at 30°C **(E)**.

**Figure 5:**
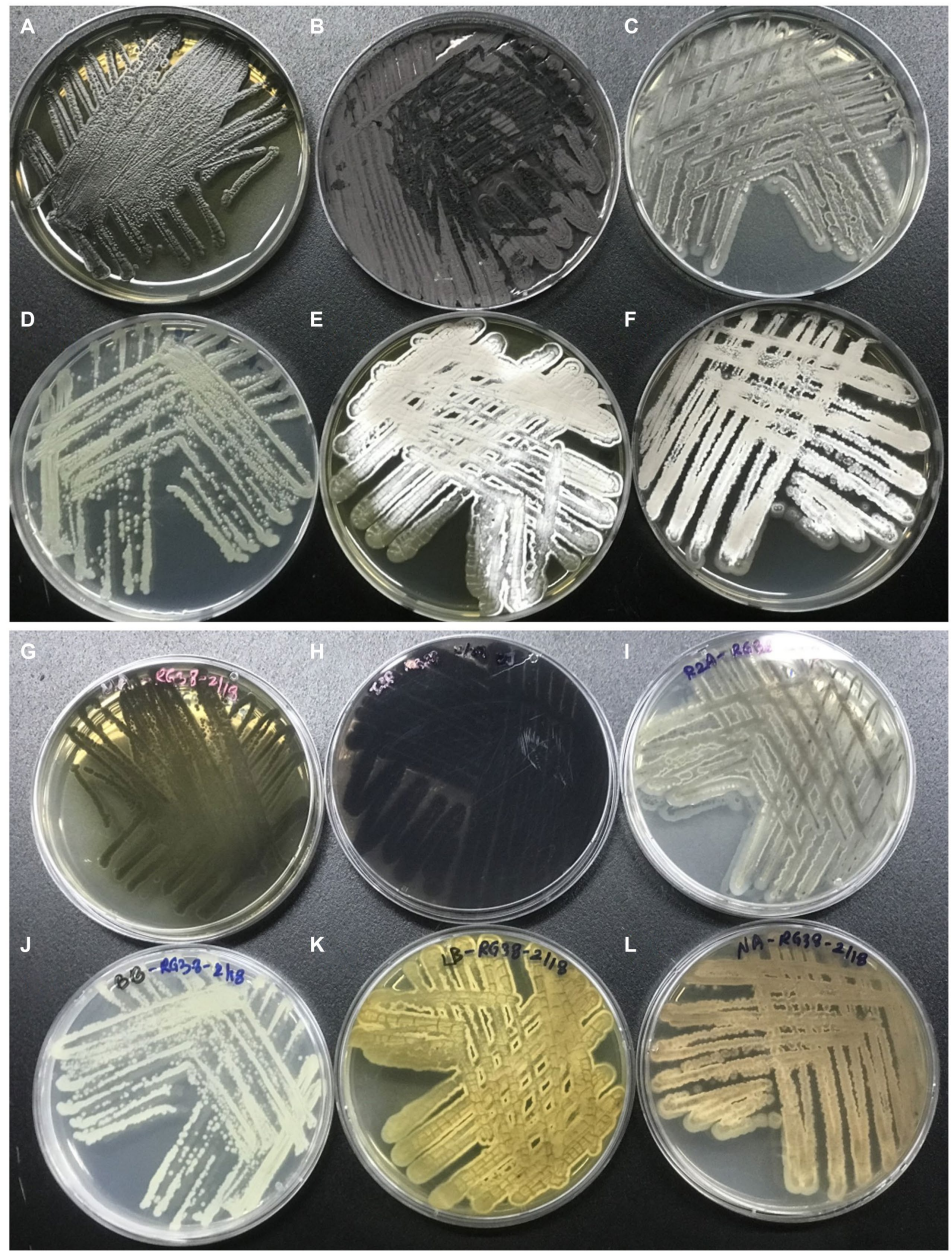
Morphology of the strain RG38^T^ in different media. Strain RG38^T^ from above in MA **(A)**, ISP2 **(B)**, R2A **(C)**, BA **(D)**, LB **(E)**, and NA **(F)**. Strain RG38^T^ from bottom in MA **(G)**, ISP2 **(H)**, R2A **(I)**, BA **(J)**, LB **(K)**, and NA **(L)**.

The phenotypic morphology of the culture was monitored after 1 week of incubation at 30°C (Figures 4A,B). Cells of the strain RG38^T^ are gram-positive, forming black melanin only on ISP2 medium or broth (Figures 4C,D). Strain RG38^T^ showed sporulation under solid culture conditions similar to those used for other *Streptomyces* and formed straight hyphae that were 0.45–0.75 μm wide with occasional single ramification on their side. Spores of the strain RG38^T^ were short cylinders of length 0.5–2.0 μm (Figure 4E). The pigmentation of both aerial and substrate mycelium was influenced by culture medium used (Figure 5). Cells exhibited robust growth across all media utilized in this study (ISP2, BA, NA, TSA, R2A, and LB). Cells grow at 10–45°C (optimum 28–30°C), at pH 5.0–10.0 (optimum, 7.0), and at 0–15% NaCl (w/v) tolerance (optimum, 0%). Strain RG38^T^ showed growth at 45°C and can tolerate upto 15% NaCl, however its close strains have not shown such characteristics. Cells are positive for catalase and oxidase. Hydrolysis of Tween 20, 40, 80, starch, casein, urease, and esculin was occurred but CM cellulose, chitin, and gelatin were not hydrolyzed. Additional differentiating biochemical characteristics of the strain RG38^T^, as compared with its close relatives, are presented in Table 3.

**Table 3:** Differential characteristics of the strain RG38^T^ in comparison to closely related species of *Streptomyces* species.

Characteristics	1	2	3	4
Isolation source	Roots	Soil	Soil	Soil
Morphology and pigmentation:				
Color of spore mass (ISP2)	Light gray	White	Olive green	Blue gray
Diffusible pigment on ISP2	+	−	+	
Growth with/at:				
15% (w/v) NaCl tolerance	+	−	−	−
pH5	+	+	−	−
45°C	+	−	−	−
Optimal temperature	25–30	28–30	28–30	28
Nitrate reduction	+	+	−	+
Indole production on tryptophan	+	−	+	+
Glucose fermentation	−	+	+	−
Arginine dihydrolase	−	+	−	−
Urease	+	+	−	−
Esculin hydrolysis	+	−	−	+
Gelatin hydrolysis	−	+	+	−
Assimilation of (20NE):				
D-Mannitol	−	+	+	−
D-Maltose	−	+	+	−
Potassium gluconate	+	−	+	+
Capric acid	−	−	−	+
Adipic acid	−	+	+	−
Malic acid	−	+	+	+
Trisodium citrate	−	+	−	+
API ZYM activities:				
Alkaline phosphatase	+	+	+	−
Lipase (C14)	+	+	+	−
Leucine arylamidase	−	+	+	+
Valline arylamidase	−	+	+	+
Cystine arylamidase	−	+	−	+
Trypsin	+	+	−	+
*α*-chymotrypsin	+	+	−	−
Acid phosphatase	+	−	+	−

Strain: 1, RG38^T^; 2, S. collinus NBRC 12759^T^; 3, S. iakyrus NRRL ISP-5482^T^ and S. glaucescens NBRC 12774^T^. All data are from this study.

### Antimicrobial activity test

Strain RG38^T^ showed antimicrobial activity against *Staphylococcus aureus* ATCC 6538, *Bacillus subtilis* KACC 16747, *Micrococcus luteus* KACC 13377, and *S. epidermidis* KACC 13234. However, antimicrobial activity against *X. campestris* pv. *campestris* KACC 10377, *Botrytis cinerea* KACC 40573, and *Pantoea agglomerans* KACC 10054 was not found. Test was performed a minimum of three times. The observed antibiotic activity varied depending on the specific growth medium employed. No antifungal activity was detected in the strain RG38^T^. Before the extraction of compounds, it is imperative to optimize the growth medium to enhance antibiotic production. Consequently, optimization tests were conducted. Antimicrobial activity was high when the strain was cultivated in NA, LB, TSA, and MA medium (Supplementary Figure S4). Antibiotic production was not found when the strain was cultured in R2A medium. To ascertain the presence of antimicrobial activity in the fermentation broth of the strain RG38^T^, we conducted a separate analysis with the resulting supernatant, subsequently employed for inhibition studies against the aforementioned pathogenic bacteria. The fermentation broth of the strain RG38^T^ showed weak inhibition of *S. epidermidis* KACC 13234. Furthermore, inhibition of *S. aureus* ATCC, *B. subtilis* KACC 16747, and *M. luteus* KACC 13377 was almost completely absent. This disparity in inhibition profiles between solid and liquid cultures suggests that the strain RG38^T^ may produce multiple distinct antibiotics that are capable of targeting various microorganisms. Furthermore, the alteration in culture methodology appears to have affected the expression patterns of secondary metabolic pathways in the strain RG38^T^. Various strategies were employed in attempts to optimize the fermentation broth for bioactive compound production, such as adjustments to the bacteria-to-medium ratio, modifications in culture duration, temperature, and pH. Unfortunately, none of the culture conditions tested were able to yield bioactive compounds with strong inhibitory effects against the pathogenic bacteria. Therefore, for this study, we opted to utilize the agar extraction method for compound extraction while considering further investigations into optimizing broth culture conditions in future research.

**Figure 6:**
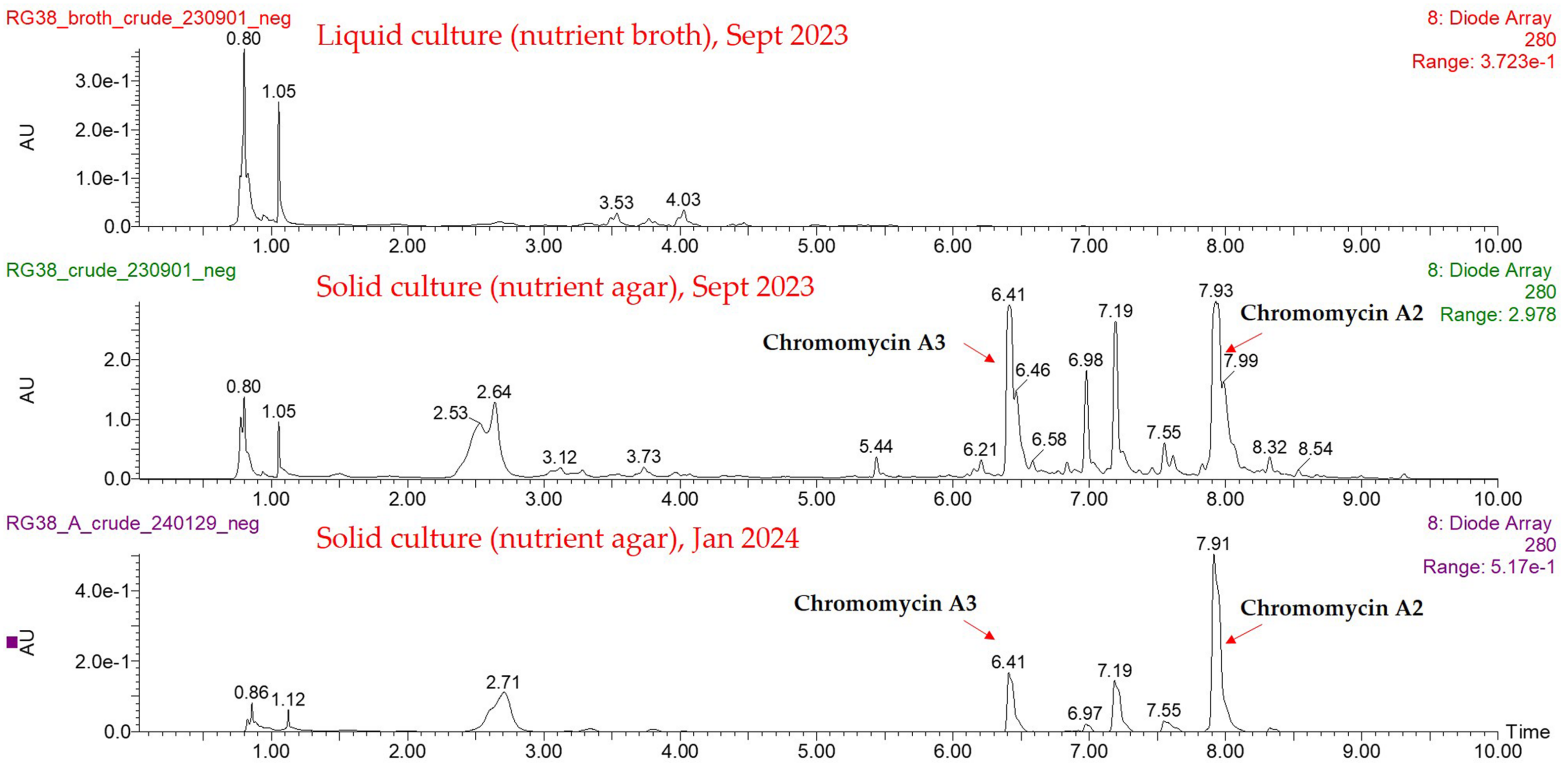
HPLC profiles of the different extracts from both liquid and solid cultures.

In addition to confirm the expression of same antibiotic production, HPLC profile was created by using different extracts from both liquid and solid cultures, where the crude extract from a solid culture condition showed the antibiotic activity only (Figure 6). Two profiles were significantly different, and the chromomycin derivatives were detected exclusively from the solid culture condition only. Additionally, we compared the HPLC profiles of two crude extracts from different time (September 2023 and January 2024). Both profiles showed that RG38^T^ consistently produced the chromomycin derivatives.

### Isolation and identification of RG38^T^ produced antimicrobial secondary metabolites through UPLC-QTOF-MS/MS

**Figure 7:**
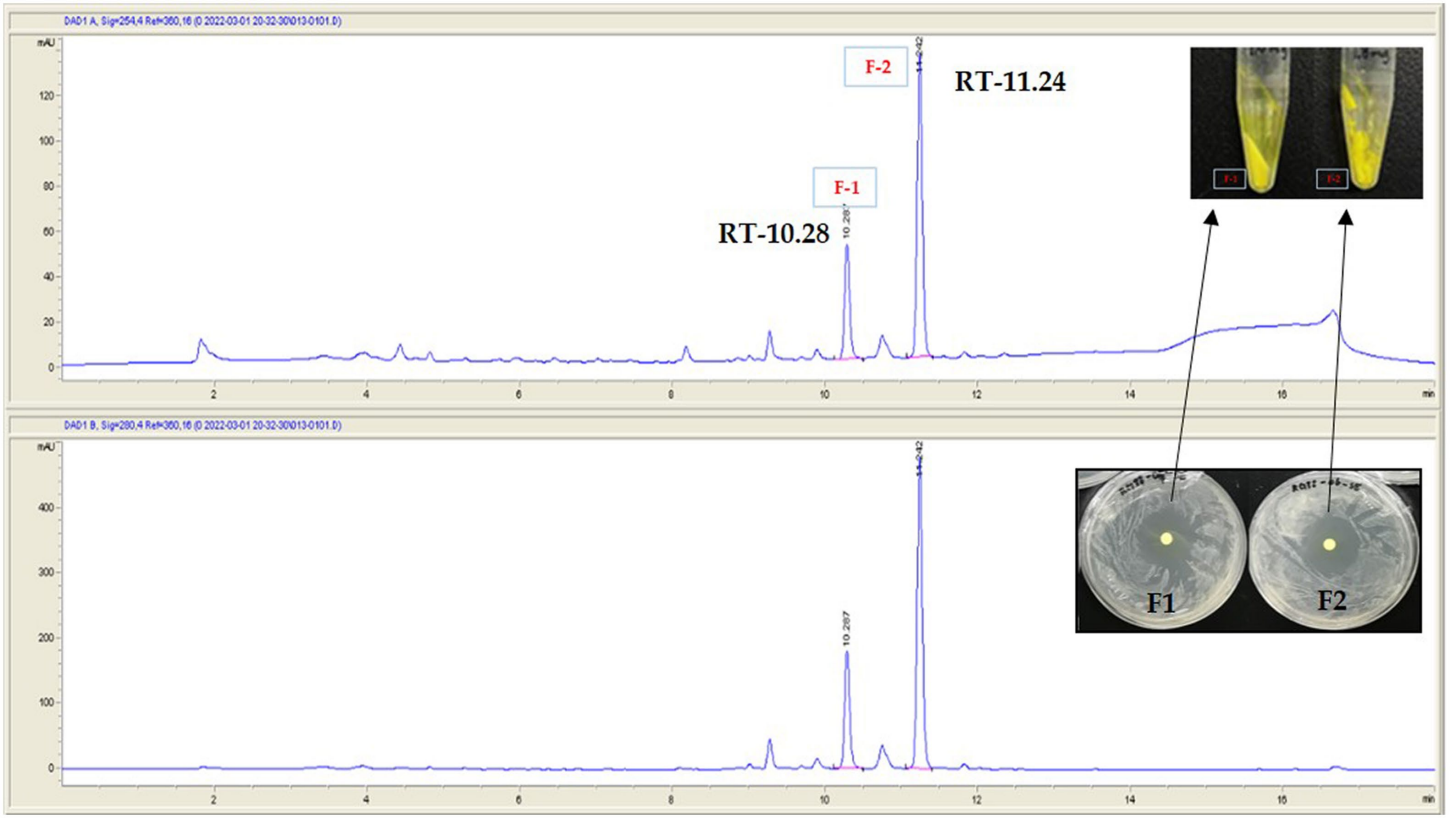
Separation of active metabolite from the crude extract of the strain RG38^T^ by using semi-preparative HPLC. Fraction-1 and fraction-2. Picture in inset is yellow powder of F-1 and F-2. RT denotes retention time.

**Figure 8:**
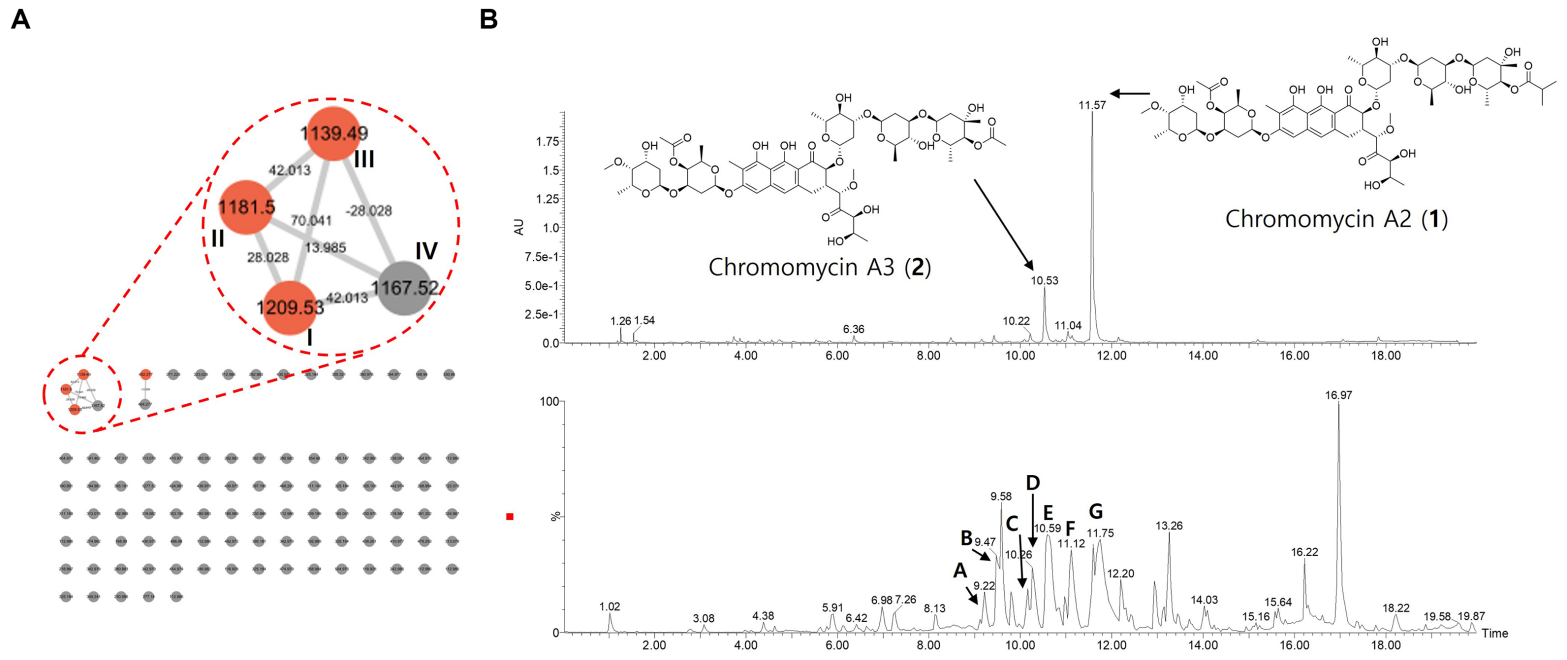
The molecular network, UV, and MS chromatograms from LC–MS/MS data of crude extract. **(A)** Molecular family of aureolic acid derivatives was annotated based on the spectral library matching on MS/MS spectra. The annotated nodes were colored as red. Properties of *m/z* and mass difference were labeled on each of the nodes and edges. **(B)** UPLC-UV chromatograms (upper, 412 nm) and base peak chromatogram (lower) of crude extract. Aureolic acid family was pointed on the chromatogram.

To investigate the bioactive metabolites produced by RG38^T^, fractionation of the major components was performed using semi-preparative HPLC system. As shown in Figure 7, two prominent fractions, F-1 and F-2, were observed from the HPLC chromatogram of RG38^T^ extract at 254 and 280 nm, respectively. The antimicrobial activities of two obtained yellow color fractions were evaluated against *Staphylococcus epidermis* KACC 13234 (Figure 8), *Staphylococcus aureus* ATCC 6538 (not shown), *Micrococcus luteus* KACC 13377 (not shown), and *Bacillus subtilis* KACC 16747 (not shown), and both fractions exhibited strong antimicrobial activities for all of them. By comparing the NMR spectra of F-1 and F-2 with the literature, the obtained fractions were identified as chromomycin A2 (1) and A3 (2), respectively (Toume et al., 2014). In this study, the NMR spectra obtained from the fractions exactly matched and aligned with the NMR spectra that were reported in previous articles (Supplementary Figures S5–S8). Detail of chemical shift is presented in Supplementary Table S1. Typically, members of the aureolic acid family, including chromomycin derivatives, show antimicrobial activity against gram-positive bacteria (Cho et al., 2020). Therefore, this study suggested that the antimicrobial activities of the RG38^T^ extract originated from the chromomycin A2 (1) and A3 (2), which are produced by RG38^T^.

In addition, we analyze the secondary metabolites derived from the strain RG38^T^ to understand its chemical diversity. Thus, the MS/MS molecular network analysis was performed with the crude extract (Figure 8A). The result showed one molecular family in the range of *m/z* 1,100–1,200. Three red nodes in the family were annotated as aureolic acid derivatives by matching MS/MS fragmentation patterns with the GNPS spectral library. In detail, the metabolites involved in the molecular family shared a same fragment ion of *m/z* 269 derived from a tricyclic core of aureolic acid family, which supported the annotation results. For example, the molecule ion with *m/z* of 1167.5 shows a loss of 42 Da from chromomycin derivatives through deacetylation or demethylation process.

Those results were identical to the genomic analysis result of RG38^T^. Combining those results, major chromatographic peaks and several peaks with similar UV absorbance patterns and MS/MS fragmentation patterns were tentatively annotated in a scaffold level to describe the chemo diversity of RG38^T^-derived metabolites (Figure 8B and Table 4).

**Table 4:** Chromatographic peaks of aureolic acid family in the LC–MS/MS profile of the strain RG38^T^_._

Peak	t_R_ (min)	Precursor ion *m/z* ([M-H]^−^)	Molecular formula	Error (ppm)	MS/MS fragments	Compounds
A	9.22	1139.4943	C_55_H_80_O_25_	2.9	991, 544, 341, 269	Deacetyl- chromomycin A3
B	9.47	1139.4927	C_55_H_80_O_25_	1.5	991, 675, 586, 341, 269	Deacetyl-chromomycin A3
C	10.16	1167.5295	C_57_H_84_O_25_	6.2	1,019, 745, 544, 341, 269	Deacetyl-chromomycin A2
D	10.26	1167.5228	C_57_H_84_O_25_	0.4	1,019, 745, 544, 341, 269	Deacetyl-chromomycin A2
E	10.59	1181.5090	C_57_H_82_O_26_	6.3	1,033, 717, 586, 341, 269	Chromomycin A3 (2)
F	11.12	1195.5192	C_58_H_84_O_26_	1.6	1,047, 731, 586, 572, 341, 269	Demethyl chromomycin A2
G	11.54	1209.5333	C_59_H_86_O_26_	0.3	1,061, 586, 341, 269	Chromomycin A2 (1)

## Conclusion

The current study focused on the isolation and characterization of beneficial microbes from previously unexplored habitats in South Korea with the aim of identifying novel bacteria possessing multifunctional properties. In this study, we isolated a novel species of *Streptomyces* which was capable of producing bioactive compounds that demonstrated substantial activity against gram-positive bacteria such as *S. aureus* ATCC 6538, *S. epidermis* KACC 13234, *M. luteus* KACC 13377, and *B. subtilis* KACC 16747. These activities served as the impetus for our efforts to isolate the bioactive molecules, elucidate their structures, and evaluate their bioactivity.

The phylogenetically most closely related *S. collinus* NBRC 12759^T^, which is known for producing the narrow-spectrum antibiotic kirromycin, exhibits activity against certain bacterial pathogens and the malaria parasite *Plasmodium falciparum* (Laiple et al., 2009). In this study, we focused on identifying antibiotics produced by the strain RG38^T^. Initially, we purified compounds that were active against different gram-positive pathogens from the solid media cultures of this isolate and elucidated their chemical structures through NMR analysis. The isolated compounds were identified as chromomycin A2 (1) and A3 (2) along with their derivatives. In addition, tandem mass spectrometry-based metabolomic approach deduced that the strain RG38^T^ is able to produce deacetyl- and demethyl-chromomycin derivatives.

Moreover, BGC of the strain RG38^T^ harbored two gene clusters of chromomycin A3 (100%), which is a known antitumor drug (Menéndez et al., 2004). Previous studies have revealed the anticancer properties of many *Streptomyces* (Parkin, 2001; Vijayabharathi et al., 2011). Well-known antitumor compounds produced by species of *Streptomyces* and are used in human chemotherapy includes, mitomycin, actinomycin, anthracycline, bleomycin, aureolic acid families, pentostatin, and resistomycin (Da Rocha et al., 2001; Aftab et al., 2015). Although *Streptomyces* strains often possess multiple BGCs, most clusters remain cryptic and inactive under normal laboratory fermentation conditions. It is, therefore, imperative to cultivate these organisms under conditions that facilitate the production of desired metabolites. Therefore, proper optimization of the novel strain RG38^T^ for antibiotic production could lead to discovery of novel bioactive product in the future.

This study also revealed that the marigold roots from which the strain RG38^T^ was isolated is a potent ecological niche characterized by unique strain diversity yet to be discovered, especially in South Korea. In summary, this distinctive habitat warrants continuous exploration for the extraction of bioactive compounds. Marigold plants themselves have been used for medicinal purposes to treat various diseases for ages in remote areas of Nepal, India, and other Asian countries. To the best of our knowledge, this study represents the first instance of isolating antimicrobial agent producing *Streptomyces* species from the marigold plant roots. Further investigations are necessary to explore the associated endophytic bacterial population of the host plant which may prove valuable in a manner similar to rhizospheric bacteria. Our findings underscore the importance of further exploring the roots of medicinal plants such as marigold as a rich source of novel metabolites with relevance to biotechnological applications.

The analysis of the16S rRNA gene sequence and whole genome of the strain RG38^T^ revealed that the designated strain belongs to the genus *Streptomyces*. Notably, this strain demonstrates melanin production when they cultivated in ISP2 medium or broth, a trait well known for its antioxidant and anti-cancer properties. Recently, this black pigment garnered attention in the field of biotechnology due to its various advantageous characteristics, such as UV-absorbing properties, suitability as a drug carrier, cation exchange capabilities, X-ray absorption capacity, and its role as amorphous semiconductors (El-Naggar and El-Ewasy, 2017). Previous studies have shown that only a limited number of actinomycetes from diverse ecological niches exhibit the ability to synthesize melanin (El-Naggar and El-Ewasy, 2017; Martínez et al., 2019). Therefore, the further study about the melanin production by the novel strain RG38^T^ is under consideration for further exploration.

### Description of *Streptomyces tagetis* sp. nov

*Streptomyces tagetis* sp. nov. (ta.ge’tis. N.L. gen. n. *tagetis*, of *Tagetes*, the plant from which the type strain was isolated).

Strain RG38^T^ is gram-positive, aerobic, non-motile actinobacterium that forms branched substrate and aerial mycelium which differentiates into short cylindrical shaped spore chains at maturity. The colors of the aerial and substrate mycelium are diverse and depend on the growth medium. Cells grow well on all media that used in this study (ISP2, BA, NA, TSA, R2A, and LB). Strain RG38^T^ found to grow at 10–45°C (optimum 28–30°C), at pH 4.0–10.0 (optimum, 7.0), and at 0–15% NaCl (w/v) tolerance (optimum, 0%). Cells are positive for catalase and oxidase. The strain hydrolyzes Tween 20, 40, 80, casein, urease, starch, and esculin but does not exhibit hydrolysis of CM cellulose, chitin, and gelatin. When grown in ISP2 solid agar and broth, strain RG38^T^ produces melanin pigment. This strain also has the ability to reduce nitrate and is positive for indole production, while it is negative for glucose fermentation and arginine dihydrolase activities. Although it hydrolyzes urease and esculin, it does not hydrolyze gelatin. In 20 NE, the assimilation of only β-galactosidase and potassium gluconate was found. The assimilation of rest of the components such as D-mannitol, D-maltose, capric acid, adipic acid, malic acid, trisodium citrate, and phenylacetic acid was not found. In API ZYM, the strain RG38^T^ was positive for alkaline phosphatase, lipase (C14), trypsin, α-chymotrypsin, acid phosphatase, α-galactosidase, and β-galactosidase but negative for leucine arylamidase, valline arylamidase, cystine arylamidase, *β*-glucuronidase, *α*-glucosidase, and *β*-glucosidase.

The type strain is RG38^T^ (=KCTC 49624^T^ = TBRC 15113^T^) which was isolated from the roots of marigold plant roots collected in the Ilsan region, South Korea. The DNA G + C content of the type strain is 73.3%.

## Data availability statement

The datasets presented in this study can be found in online repositories. The names of the repository/repositories and accession number(s) can be found at: the MassIVE database (https://massive.ucsd.edu/) - RG38T.

## Author contributions

GC: Conceptualization, Data curation, Formal analysis, Investigation, Methodology, Software, Validation, Visualization, Writing – original draft, Writing – review & editing. MK: Data curation, Formal analysis, Investigation, Methodology, Resources, Software, Visualization, Writing – review & editing. IK: Data curation, Formal analysis, Investigation, Resources, Validation, Writing – review & editing. DT: Data curation, Formal analysis, Investigation, Validation, Writing – review & editing. Y-WK: Data curation, Methodology, Resources, Software, Supervision, Writing – review & editing. HK: Conceptualization, Data curation, Funding acquisition, Methodology, Project administration, Resources, Software, Supervision, Validation, Writing – original draft, Writing – review & editing. TS: Conceptualization, Data curation, Funding acquisition, Investigation, Methodology, Project administration, Resources, Software, Supervision, Validation, Writing – original draft, Writing – review & editing.

## Footnotes

^1^ http://ggdc.dsmz.de

^2^ www.ezbiocloud.net/tools/ani

^3^ http://enve-omics.ce.gatech.edu/aai/

^4^ https://ecogenomics.github.io/CheckM

^5^ https://www.microbiologyresearch.org/content/journal/ijsem?page=about-journal

^6^ https://gnps.ucsd.edu

^7^ https://gnps.ucsd.edu/ProteoSAFe/status.jsp?task=14d32e0bbe0848a0a625b1786f574f02

^8^ https://gnps.ucsd.edu/ProteoSAFe/status.jsp?task=d99405b0ce184dbdba30177d8f6a324b
