# Supplementary material for: Streptomyces tagetis sp. nov., a chromomycin producing bacteria isolated from the roots of Tagetes patula
Source: Front Microbiol. 2024 Mar 1;15:1361583. doi: 10.3389/fmicb.2024.1361583 (PMC10940327; doi:10.3389/fmicb.2024.1361583)

***Streptomyces tagetis* sp. nov., a Chromomycin producing bacteria isolated from the roots of *Tagetes patula***

Author names

Geeta Chhetri^1^, Myung Ji Kim^2^, Inhyup Kim^1^, Duc V. H. Tran^2^, Young-Woo Kim^2^, Hyun Woo Kim^2, *^ and Taegun Seo^1, *^

**Affiliation**

^1^Department of Life Science, Dongguk University-Seoul, Goyang 10326, South Korea

**^2^** College of Pharmacy and Integrated Research Institute for Drug Development, Dongguk University-Seoul, Goyang 10326, South Korea

***Correspondence:**

Corresponding Authors:

Hyun Woo Kim

hwkim8906@dongguk.edu

Tel: +82-31-961-5224

Taegun Seo

tseo@dongguk.edu

Tel : +82-31-961-5135

**Supplementary Information**

**Summary:**

**Figure S1.** *Streptomyces tagetis* RG38^T^ was isolated during the characterization of bacteria present in the roots of a marigold plants. Samples were collected from the garden of Dongguk University, Ilsan, Republic of Korea (37° 40’ 26.4” N 126° 48’ 20.88” E).

**Figure S2.** Maximum-likelihood tree based on 16S rRNA gene sequences showing the relationship between strain RG38^T^ and related species. Bootstrap values (based on 1000 replications) greater than 50% are shown at branch points. Bar, 0.020 substitutions per nucleotide position.

**Figure S3.** A maximum-parsimony tree based on 16S rRNA gene sequences, showing the phylogenetic position of strain RG38^T^ and other closely related members. Numbers at nodes are levels of bootstrap support (>50%) based on 1000 resamplings. *Pontibacter* oryzae KIRAN^T^(MH165268) was used as an out-group. GeneBank accession numbers are given in parenthesis. Bar, 50 substitutions per nucleotide position.

**Figure S4.** Strain RG38^T^ was spotted after grown in different media to identify optimum media for strong antimicrobial activity. Prior to this *Staphylococcus aureus* ATCC 6538 (A), *Bacillus subtilis* KACC 16747 (B) and *Staphylococcus epidermidis* KACC 13234 (C) were spread over three different R2A agar plates.

**Figure S5.** ^1^H NMR spectrum (400 MHz) of compound **1** in CDCl_3_

**Figure S6.** ^13^C NMR spectrum (100 MHz) of compound **1** in CDCl_3_

**Figure S7.** ^1^H NMR spectrum (400 MHz) of compound **2** in CDCl_3_

**Figure S8.** ^13^C NMR spectrum (100 MHz) of compound **2** in CDCl_3_

**Figure S1.** *Streptomyces tagetis* RG38^T^ was isolated during the characterization of bacteria present in the roots of a marigold plants. Samples were collected from the garden of Dongguk University, Ilsan, Republic of Korea (37° 40’ 26.4” N 126° 48’ 20.88” E).


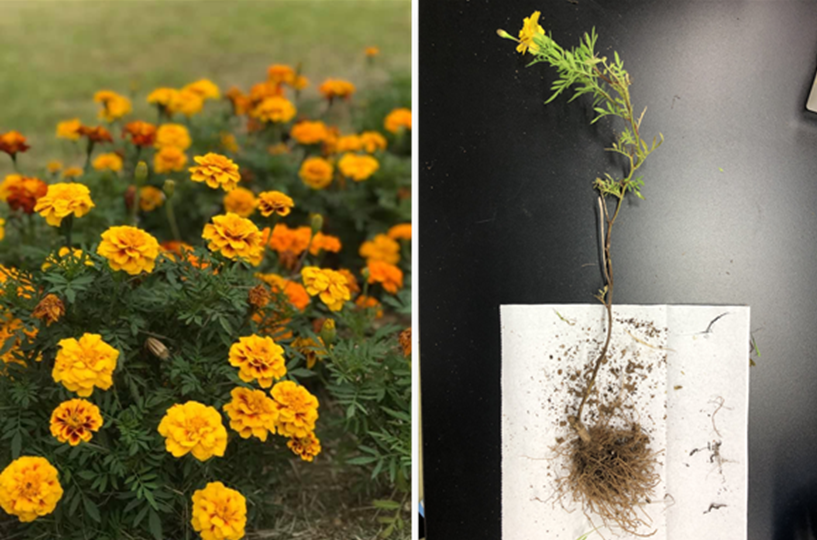


**Figure S2.** A maximum-likelihood tree based on 16S rRNA gene sequences, showing the phylogenetic position of strain RG38^T^ and other closely related members. Numbers at nodes are levels of bootstrap support based on 1000 resamplings. *Pontibacter oryzae* KIRAN^T^(MH165268) was used as an out-group. GeneBank accession numbers are given in parenthesis. Bar, 0.020 substitutions per nucleotide position.


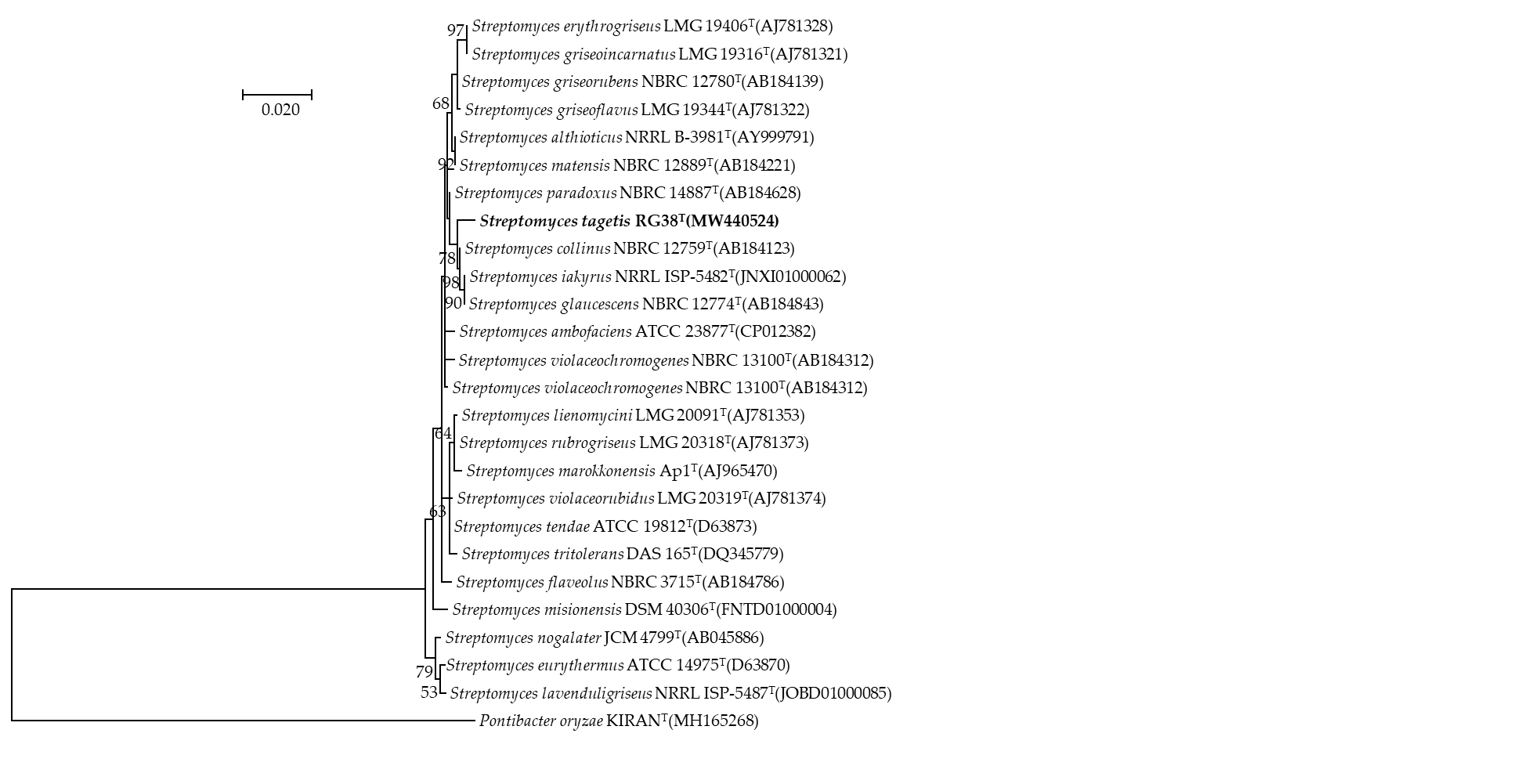


**Figure S3.** A maximum-parsimony tree based on 16S rRNA gene sequences, showing the phylogenetic position of strain RG38^T^ and other closely related members. Numbers at nodes are levels of bootstrap support based on 1000 resamplings. *Pontibacter oryzae* KIRAN^T^(MH165268) was used as an out-group. GeneBank accession numbers are given in parenthesis. Bar, 50 substitutions per nucleotide position.

##
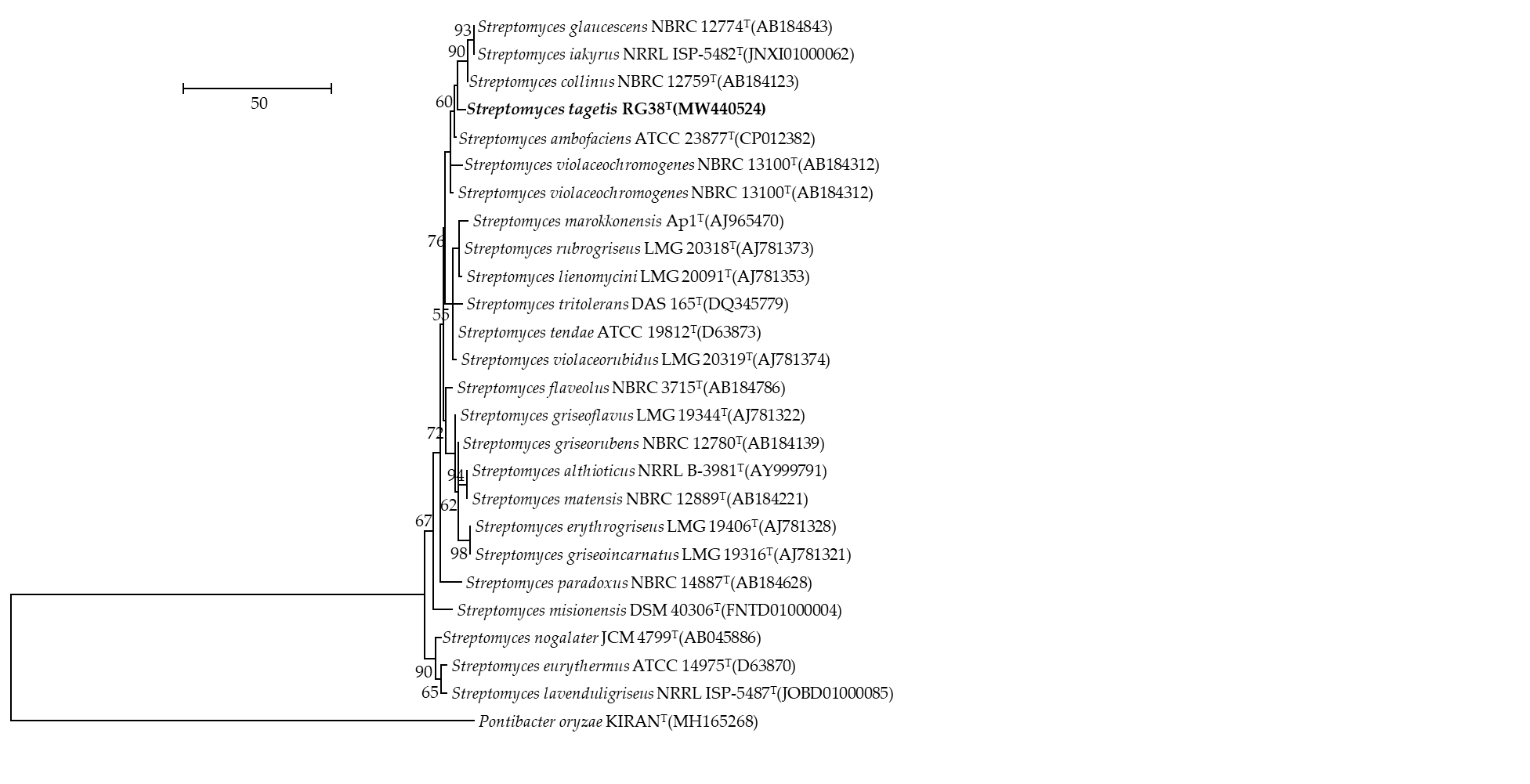


**Figure S4.** Strain RG38^T^ was spotted after grown in different media to identify optimum media for strong antimicrobial activity. Prior to this *Staphylococcus aureus* ATCC 6538 (A), *Bacillus subtilis* KACC 16747 (B) and *Staphylococcus epidermidis* KACC 13234 (C) were spread over three different R2A agar plates.

**
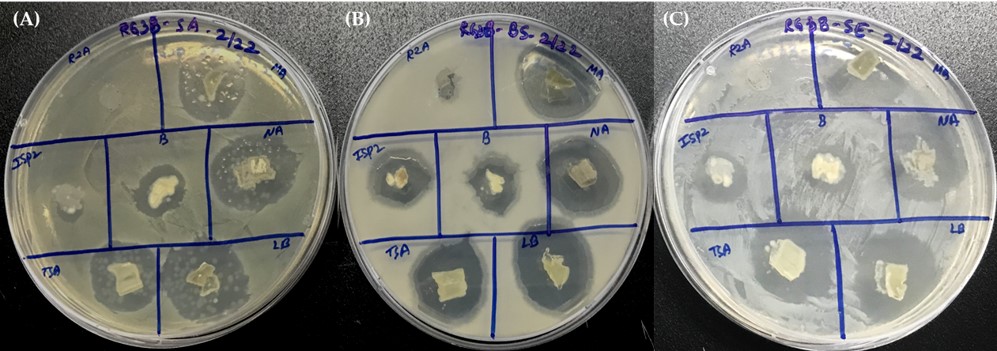
**

**Figure S5.** ^1^H NMR spectrum (400 MHz) of compound **1** in CDCl_3_


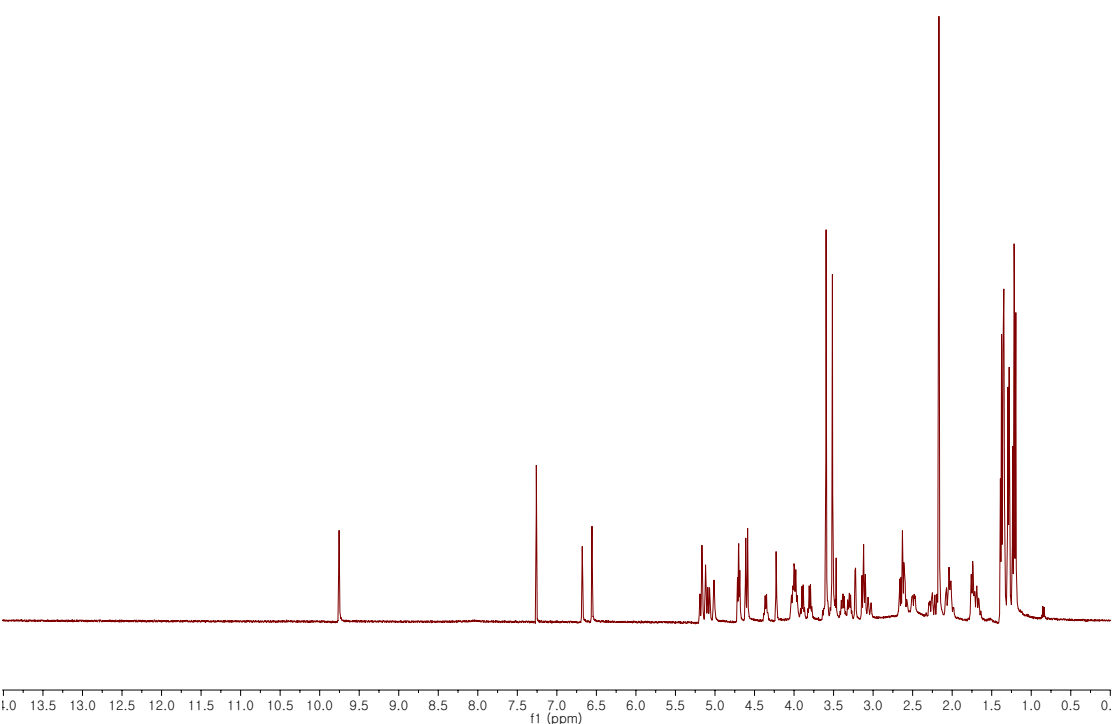


**Figure S6.** ^13^C NMR spectrum (100 MHz) of compound **1** in CDCl_3_


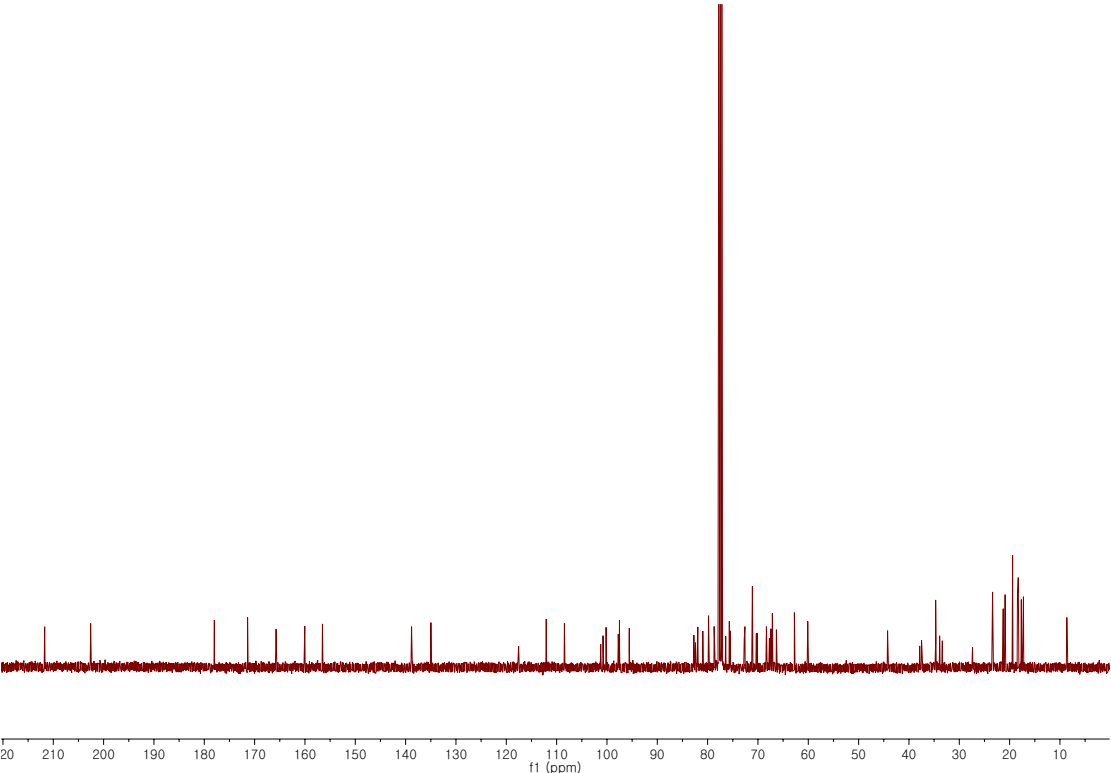


**Figure S7.** ^1^H NMR spectrum (400 MHz) of compound **2** in CDCl_3_


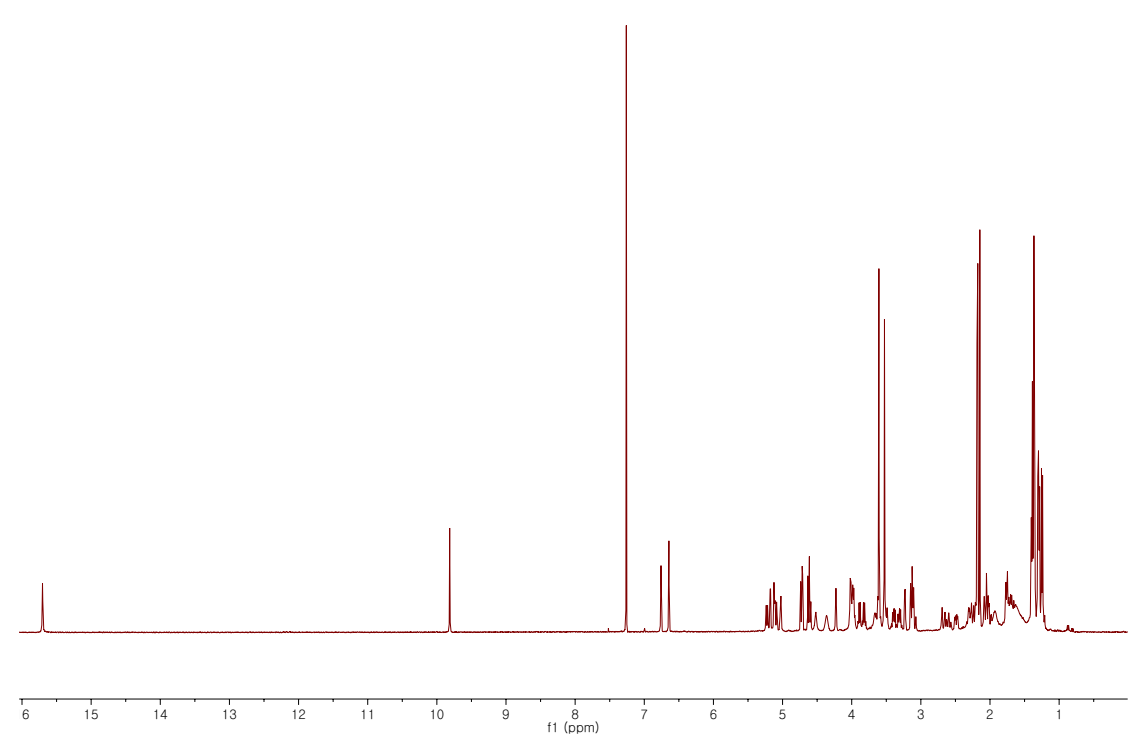


**Figure S8.** ^13^C NMR spectrum (100 MHz) of compound **2** in CDCl_3_


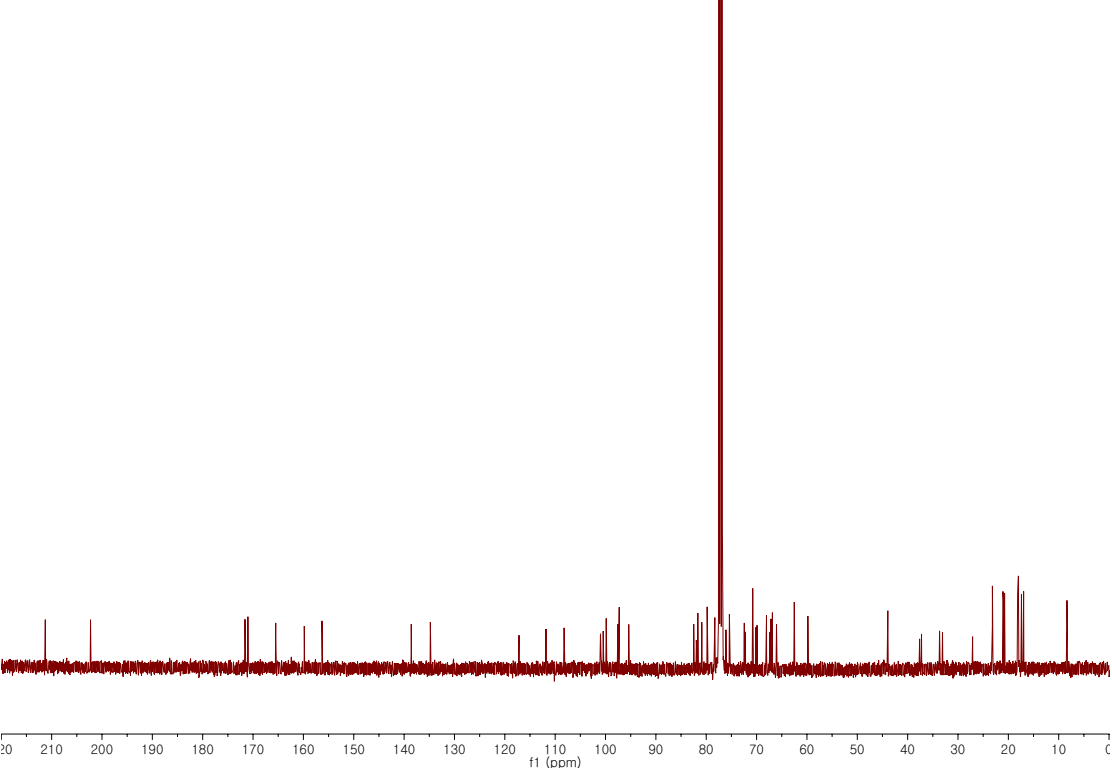


**Table S1.** Detail chemical shift of NMR spectra.

|  | **Chromomycin A3 (1)** | **Chromomycin A2 (2)** |
| --- | --- | --- |
| **Aglycone** |  |  |
| C-1 | 202.3 | 202.3 |
| C-2 | 76.1 | 76.1 |
| C-3 | 43.9 | 43.8 |
| C-4 | 27.1 | 27.1 |
| C-4a | 134.8 | 134.7 |
| C-5 | 101.0 | 100.9 |
| C-6 | 159.8 | 159.7 |
| C-7 | 111.8 | 111.7 |
| C-8 | 165.5 | 165.4 |
| C-8a | 108.3 | 108.1 |
| C-9 | 156.3 | 156.2 |
| C-9a | 108.2 | 108.1 |
| C-10 | 117.2 | 117.2 |
| C-10a | 138.6 | 138.5 |
| 7-CH3 | 8.4 | 8.3 |
| C-1' | 82.5 | 82.4 |
| C-2' | 211.3 | 211.4 |
| C-3' | 79.8 | 79.5 |
| C-4' | 68.0 | 68.0 |
| C-5' | 20.8 | 20.6 |
| 1'-OCH3 | 59.8 | 59.8 |
| **D-Chromose A** |  |  |
| C-1 | 95.4 | 97.2 |
| C-2 | 33.1 | 33.1 |
| C-3 | 66.0 | 66.0 |
| C-4 | 82.0 | 82.1 |
| C-5 | 67.2 | 67.1 |
| C-6 | 17.4 | 17.4 |
| OCH3 | 62.5 | 62.4 |
| **L-Chromose B** |  |  |
| C-1 | 97.3 | 95.2 |
| C-2 | 43.9 | 43.9 |
| C-3 | 70.1 | 70.0 |
| C-4 | 80.9 | 80.6 |
| C-5 | 66.9 | 66.8 |
| C-6 | 18.0 | 18.0 |
| 3-CH3 | 23.2 | 23.1 |
| CH3C | 21.1 | 19.1 |
| CH3C |  | 19.1 |
| CH3CHCO |  | 34.5 |
| CO | 171.0 | 171.1 |
| **D-Chromose C** |  |  |
| C-1 | 100.5 | 100.5 |
| C-2 | 37.6 | 37.6 |
| C-3 | 81.7 | 81.6 |
| C-4 | 75.4 | 75.4 |
| C-5 | 72.3 | 72.3 |
| C-6 | 18.2 | 18.1 |
| **D-Chromose C'** |  |  |
| C-1 | 99.9 | 99.9 |
| C-2 | 37.2 | 37.2 |
| C-3 | 78.3 | 78.4 |
| C-4 | 75.3 | 75.2 |
| C-5 | 72.4 | 72.4 |
| C-6 | 18.0 | 18.0 |
| **D-Chromose D** |  |  |
| C-1 | 97.6 | 97.4 |
| C-2 | 33.7 | 33.6 |
| C-3 | 70.8 | 70.8 |
| C-4 | 67.4 | 67.4 |
| C-5 | 69.9 | 69.8 |
| C-6 | 17.0 | 16.9 |
| CH3CO | 171.6 | 177.7 |
| CH3CO | 21.0 | 21.0 |

##
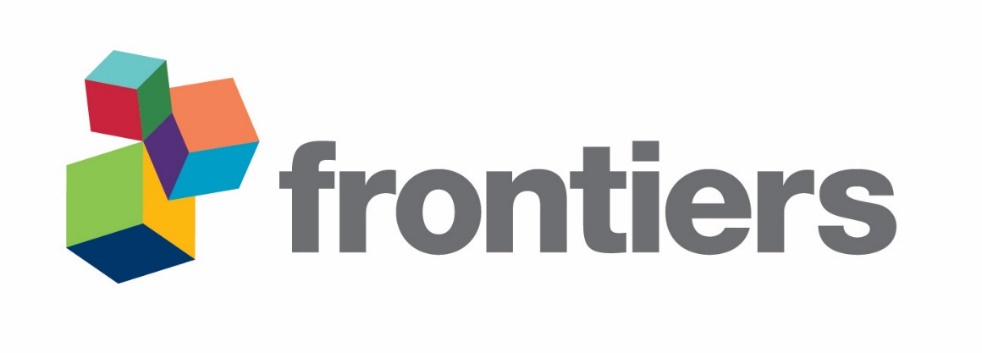

Supplement: Supplementary file 1 [file Data_Sheet_1.docx]
